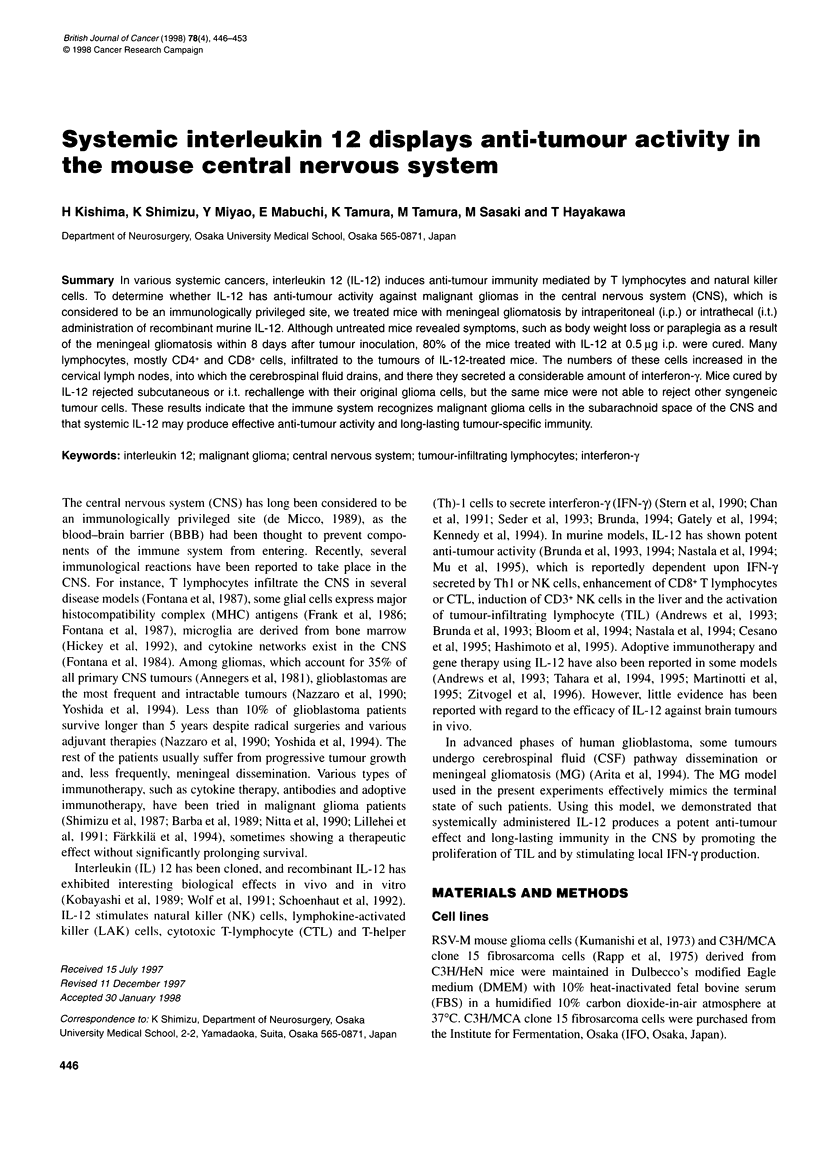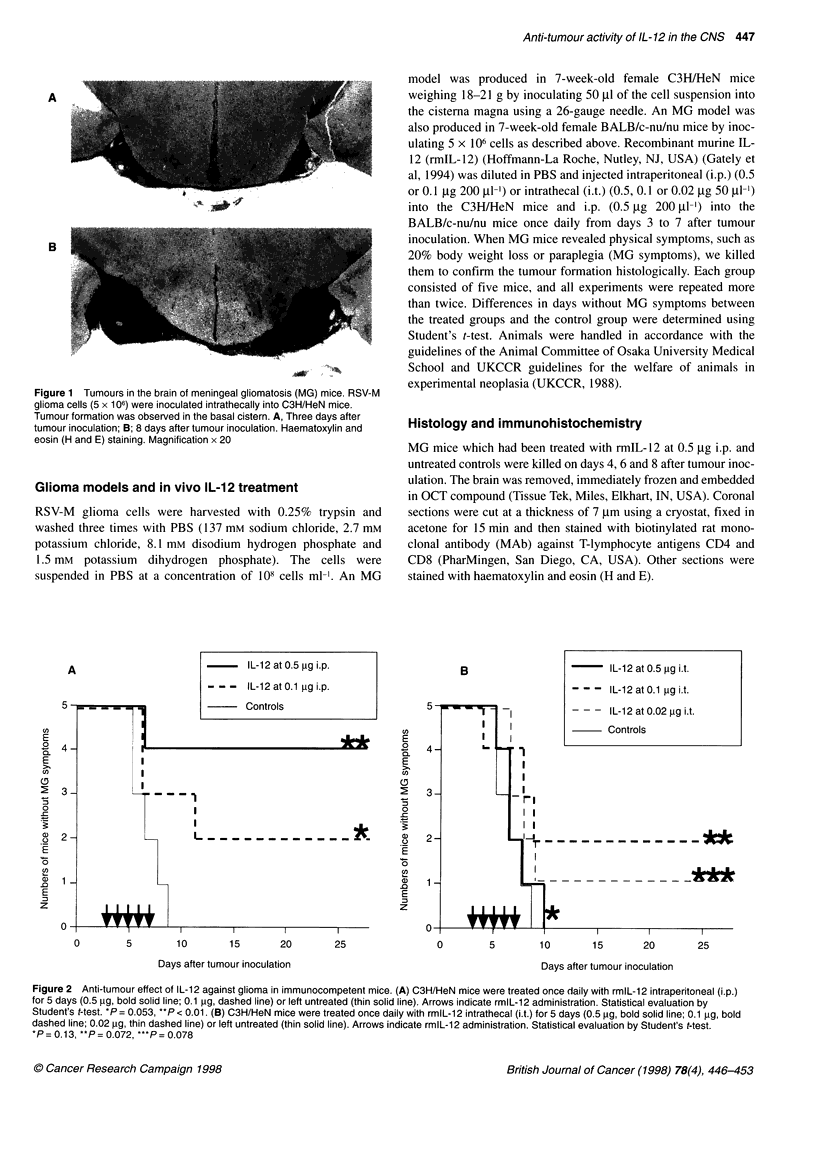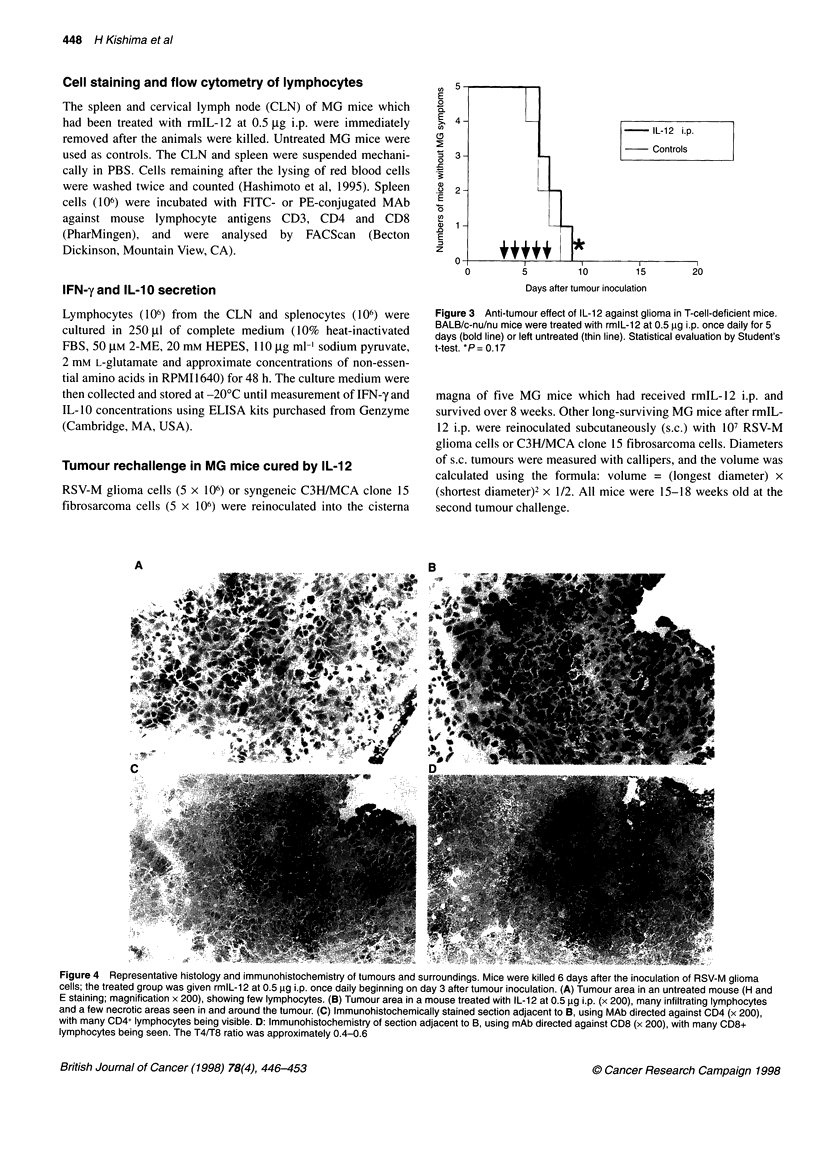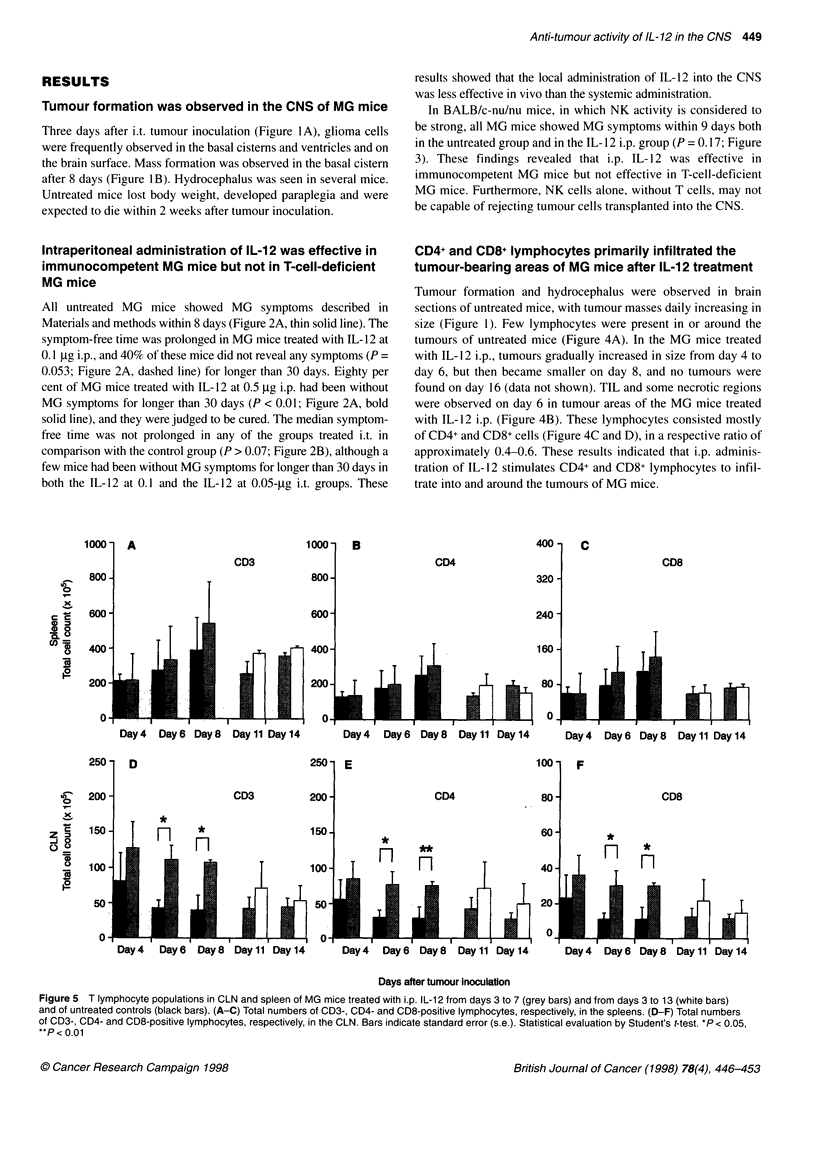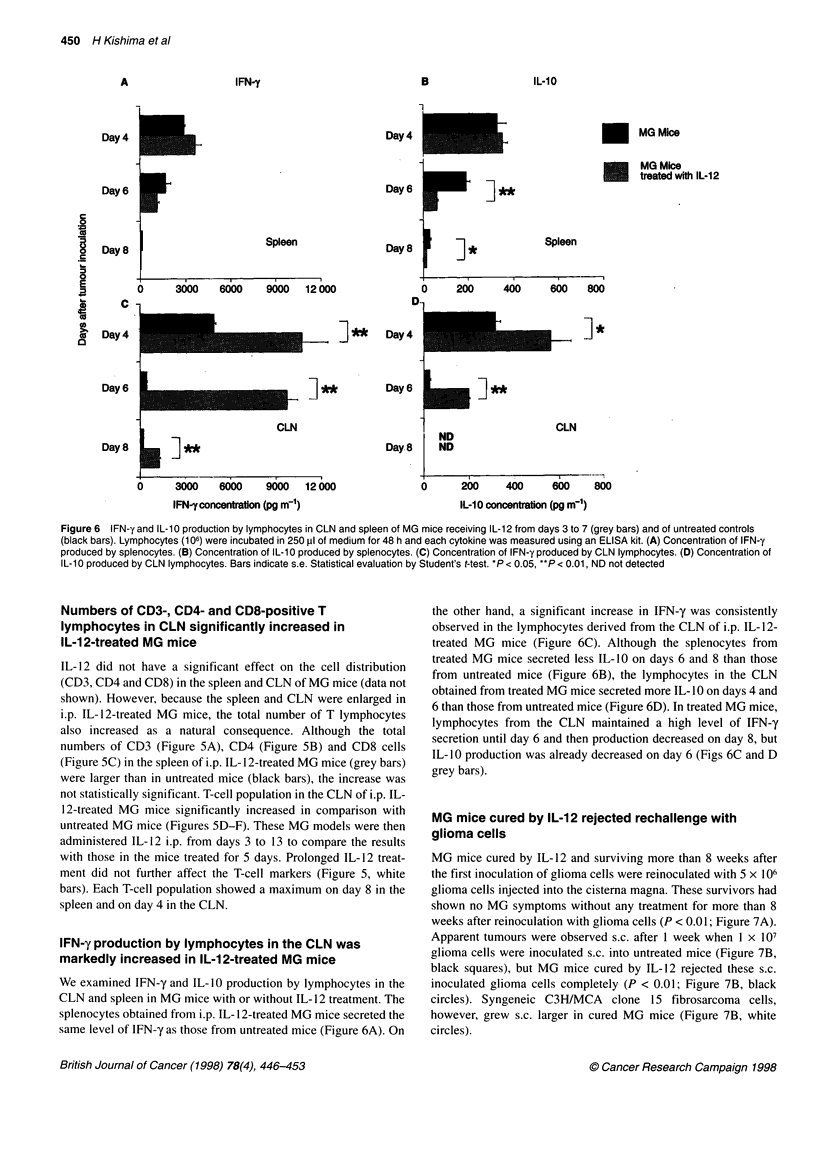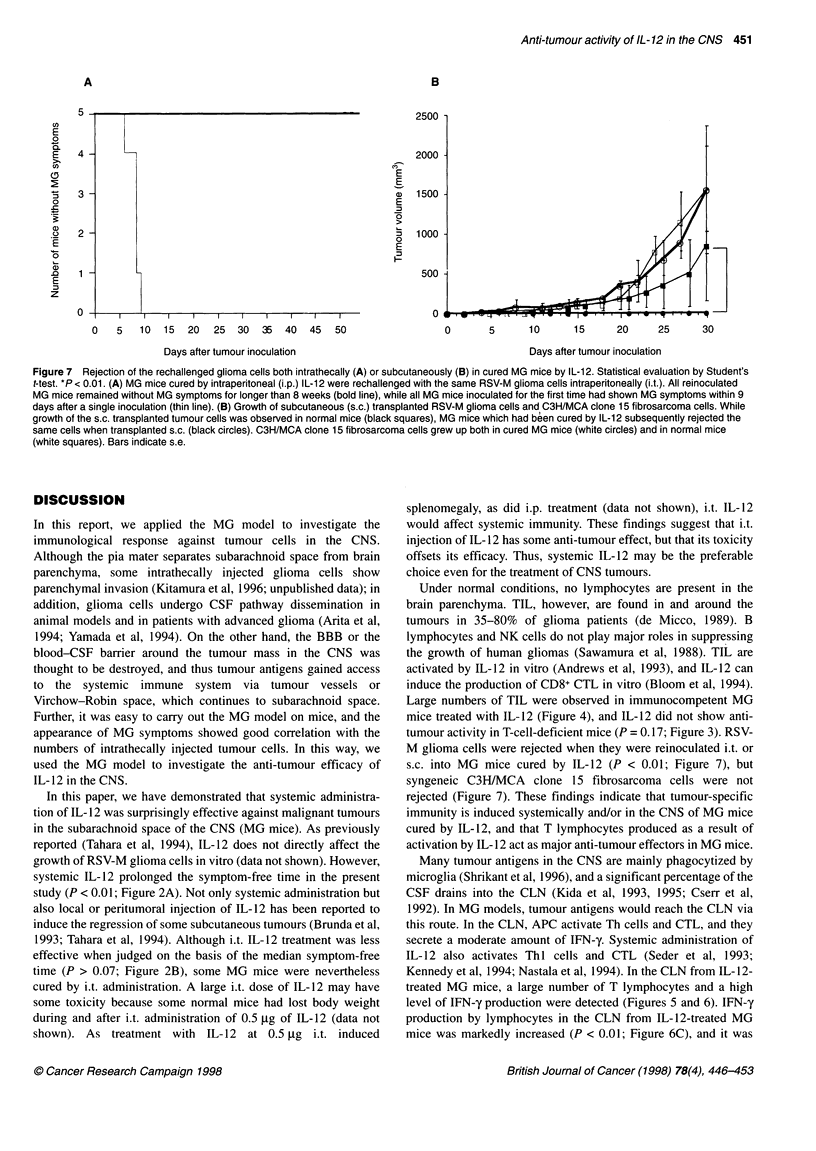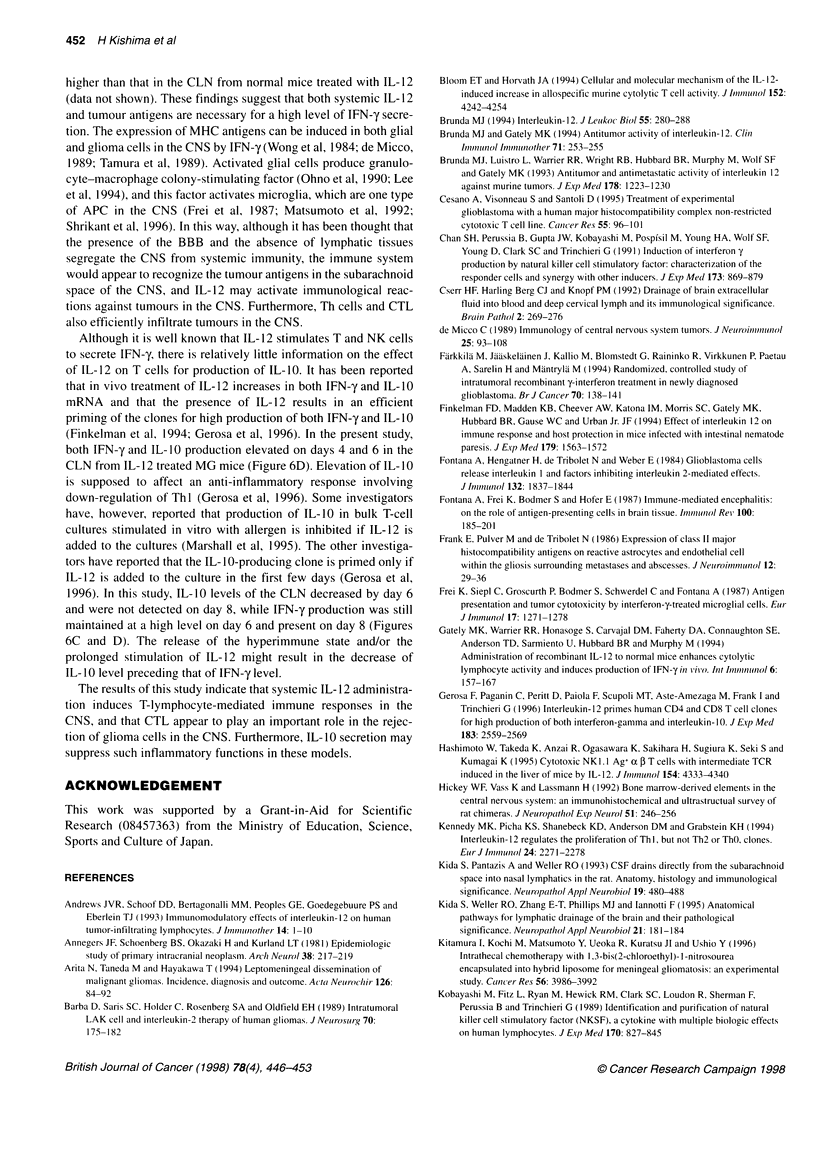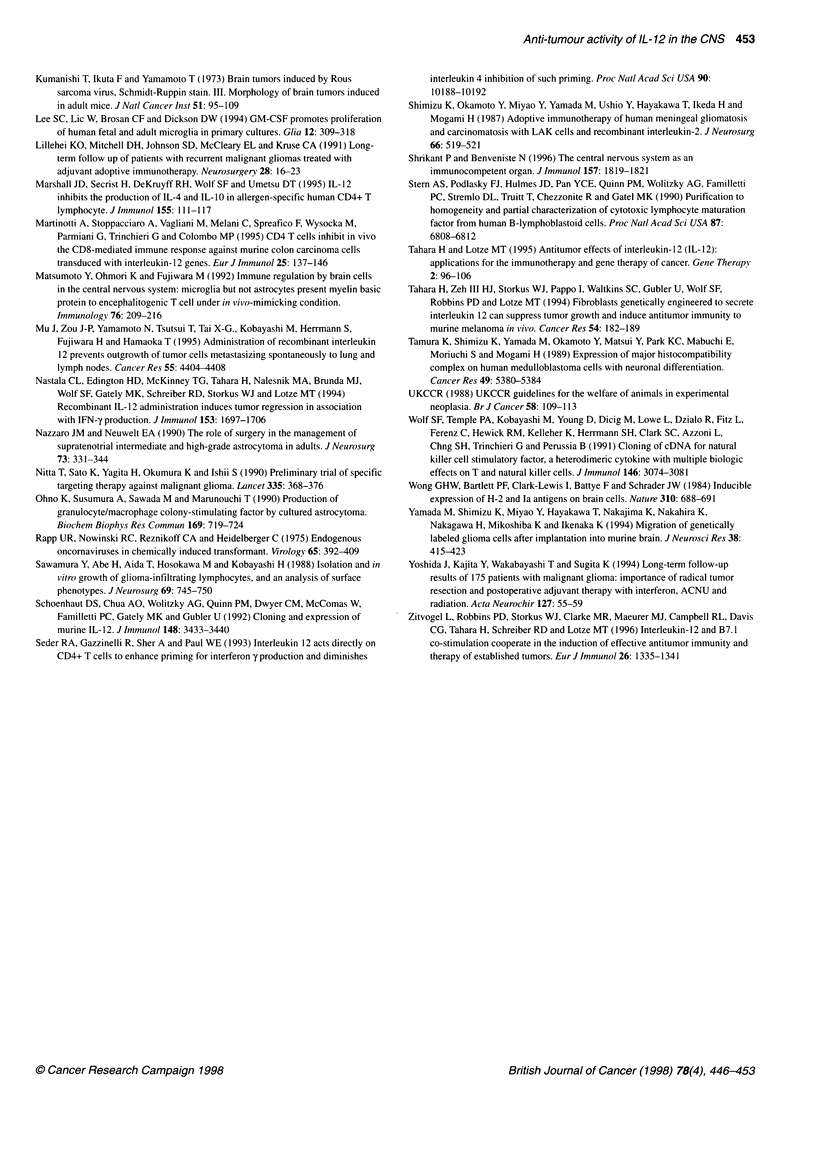# Systemic interleukin 12 displays anti-tumour activity in the mouse central nervous system.

**DOI:** 10.1038/bjc.1998.513

**Published:** 1998-08

**Authors:** H. Kishima, K. Shimizu, Y. Miyao, E. Mabuchi, K. Tamura, M. Tamura, M. Sasaki, T. Hakakawa

**Affiliations:** Department of Neurosurgery, Osaka University Medical School, Suita, Japan.

## Abstract

**Images:**


British Journal of Cancer (1998) 78(4), 446–453
© 1998 Cancer Research Campaign

### Systemic interleukin 12 displays anti-tumour activity in the mouse central nervous system

**H Kishima, K Shimizu, Y Miyao, E Mabuchi, K Tamura, M Tamura, M Sasaki and T Hayakawa**

Department of Neurosurgery, Osaka University Medical School, Osaka 565-0871, Japan

**Summary** In various systemic cancers, interleukin 12 (IL-12) induces anti-tumour immunity mediated by T lymphocytes and natural killer cells. To determine whether IL-12 has anti-tumour activity against malignant gliomas in the central nervous system (CNS), which is considered to be an immunologically privileged site, we treated mice with meningeal gliomatosis by intraperitoneal (i.p.) or intrathecal (i.t.) administration of recombinant murine IL-12. Although untreated mice revealed symptoms, such as body weight loss or paraplegia as a result of the meningeal gliomatosis within 8 days after tumour inoculation, 80% of the mice treated with IL-12 at 0.5 μg i.p. were cured. Many lymphocytes, mostly $CD4^+$ and $CD8^+$ cells, infiltrated to the tumours of IL-12-treated mice. The numbers of these cells increased in the cervical lymph nodes, into which the cerebrospinal fluid drains, and there they secreted a considerable amount of interferon-γ. Mice cured by IL-12 rejected subcutaneous or i.t. rechallenge with their original glioma cells, but the same mice were not able to reject other syngeneic tumour cells. These results indicate that the immune system recognizes malignant glioma cells in the subarachnoid space of the CNS and that systemic IL-12 may produce effective anti-tumour activity and long-lasting tumour-specific immunity.

**Keywords:** interleukin 12; malignant glioma; central nervous system; tumour-infiltrating lymphocytes; interferon-γ

The central nervous system (CNS) has long been considered to be an immunologically privileged site (de Micco, 1989), as the blood–brain barrier (BBB) had been thought to prevent components of the immune system from entering. Recently, several immunological reactions have been reported to take place in the CNS. For instance, T lymphocytes infiltrate the CNS in several disease models (Fontana et al, 1987), some glial cells express major histocompatibility complex (MHC) antigens (Frank et al, 1986; Fontana et al, 1987), microglia are derived from bone marrow (Hickey et al, 1992), and cytokine networks exist in the CNS (Fontana et al, 1984). Among gliomas, which account for 35% of all primary CNS tumours (Annegers et al, 1981), glioblastomas are the most frequent and intractable tumours (Nazzaro et al, 1990; Yoshida et al, 1994). Less than 10% of glioblastoma patients survive longer than 5 years despite radical surgeries and various adjuvant therapies (Nazzaro et al, 1990; Yoshida et al, 1994). The rest of the patients usually suffer from progressive tumour growth and, less frequently, meningeal dissemination. Various types of immunotherapy, such as cytokine therapy, antibodies and adoptive immunotherapy, have been tried in malignant glioma patients (Shimizu et al, 1987; Barba et al, 1989; Nitta et al, 1990; Lillehei et al, 1991; Färkkilä et al, 1994), sometimes showing a therapeutic effect without significantly prolonging survival.

Interleukin (IL) 12 has been cloned, and recombinant IL-12 has exhibited interesting biological effects in vivo and in vitro (Kobayashi et al, 1989; Wolf et al, 1991; Schoenhaut et al, 1992). IL-12 stimulates natural killer (NK) cells, lymphokine-activated killer (LAK) cells, cytotoxic T-lymphocyte (CTL) and T-helper (Th)-1 cells to secrete interferon-γ (IFN-γ) (Stern et al, 1990; Chan et al, 1991; Seder et al, 1993; Brunda, 1994; Gately et al, 1994; Kennedy et al, 1994). In murine models, IL-12 has shown potent anti-tumour activity (Brunda et al, 1993, 1994; Nastala et al, 1994; Mu et al, 1995), which is reportedly dependent upon IFN-γ secreted by Th1 or NK cells, enhancement of $CD8^+$ T lymphocytes or CTL, induction of $CD3^+$ NK cells in the liver and the activation of tumour-infiltrating lymphocyte (TIL) (Andrews et al, 1993; Brunda et al, 1993; Bloom et al, 1994; Nastala et al, 1994; Cesano et al, 1995; Hashimoto et al, 1995). Adoptive immunotherapy and gene therapy using IL-12 have also been reported in some models (Andrews et al, 1993; Tahara et al, 1994, 1995; Martinotti et al, 1995; Zitvogel et al, 1996). However, little evidence has been reported with regard to the efficacy of IL-12 against brain tumours in vivo.

In advanced phases of human glioblastoma, some tumours undergo cerebrospinal fluid (CSF) pathway dissemination or meningeal gliomatosis (MG) (Arita et al, 1994). The MG model used in the present experiments effectively mimics the terminal state of such patients. Using this model, we demonstrated that systemically administered IL-12 produces a potent anti-tumour effect and long-lasting immunity in the CNS by promoting the proliferation of TIL and by stimulating local IFN-γ production.

### MATERIALS AND METHODS

### Cell lines

RSV-M mouse glioma cells (Kumanishi et al, 1973) and C3H/MCA clone 15 fibrosarcoma cells (Rapp et al, 1975) derived from C3H/HeN mice were maintained in Dulbecco's modified Eagle medium (DMEM) with 10% heat-inactivated fetal bovine serum (FBS) in a humidified 10% carbon dioxide-in-air atmosphere at 37°C. C3H/MCA clone 15 fibrosarcoma cells were purchased from the Institute for Fermentation, Osaka (IFO, Osaka, Japan).

Received 15 July 1997
Revised 11 December 1997
Accepted 30 January 1998

Correspondence to: K Shimizu, Department of Neurosurgery, Osaka University Medical School, 2-2, Yamadaoka, Suita, Osaka 565-0871, Japan

**Figure 1** Tumours in the brain of meningeal gliomatosis (MG) mice. RSV-M glioma cells (5 × 10⁶) were inoculated intrathecally into C3H/HeN mice. Tumour formation was observed in the basal cistern. **A**, Three days after tumour inoculation; **B**; 8 days after tumour inoculation. Haematoxylin and eosin (H and E) staining. Magnification × 20

### Glioma models and in vivo IL-12 treatment

RSV-M glioma cells were harvested with 0.25% trypsin and washed three times with PBS (137 mM sodium chloride, 2.7 mM potassium chloride, 8.1 mM disodium hydrogen phosphate and 1.5 mM potassium dihydrogen phosphate). The cells were suspended in PBS at a concentration of $10^8$ cells $ml^{-1}$. An MG model was produced in 7-week-old female C3H/HeN mice weighing 18–21 g by inoculating 50 μl of the cell suspension into the cisterna magna using a 26-gauge needle. An MG model was also produced in 7-week-old female BALB/c-nu/nu mice by inoculating $5 \times 10^6$ cells as described above. Recombinant murine IL-12 (rmIL-12) (Hoffmann-La Roche, Nutley, NJ, USA) (Gately et al, 1994) was diluted in PBS and injected intraperitoneal (i.p.) (0.5 or 0.1 μg 200 $\mu l^{-1}$) or intrathecal (i.t.) (0.5, 0.1 or 0.02 μg 50 $\mu l^{-1}$) into the C3H/HeN mice and i.p. (0.5 μg 200 $\mu l^{-1}$) into the BALB/c-nu/nu mice once daily from days 3 to 7 after tumour inoculation. When MG mice revealed physical symptoms, such as 20% body weight loss or paraplegia (MG symptoms), we killed them to confirm the tumour formation histologically. Each group consisted of five mice, and all experiments were repeated more than twice. Differences in days without MG symptoms between the treated groups and the control group were determined using Student's *t*-test. Animals were handled in accordance with the guidelines of the Animal Committee of Osaka University Medical School and UKCCR guidelines for the welfare of animals in experimental neoplasia (UKCCR, 1988).

### Histology and immunohistochemistry

MG mice which had been treated with rmIL-12 at 0.5 μg i.p. and untreated controls were killed on days 4, 6 and 8 after tumour inoculation. The brain was removed, immediately frozen and embedded in OCT compound (Tissue Tek, Miles, Elkhart, IN, USA). Coronal sections were cut at a thickness of 7 μm using a cryostat, fixed in acetone for 15 min and then stained with biotinylated rat monoclonal antibody (MAb) against T-lymphocyte antigens CD4 and CD8 (PharMingen, San Diego, CA, USA). Other sections were stained with haematoxylin and eosin (H and E).

**Figure 2** Anti-tumour effect of IL-12 against glioma in immunocompetent mice. (**A**) C3H/HeN mice were treated once daily with rmIL-12 intraperitoneal (i.p.) for 5 days (0.5 μg, bold solid line; 0.1 μg, dashed line) or left untreated (thin solid line). Arrows indicate rmIL-12 administration. Statistical evaluation by Student's *t*-test. $^*P = 0.053$, $^{**}P < 0.01$. (**B**) C3H/HeN mice were treated once daily with rmIL-12 intrathecal (i.t.) for 5 days (0.5 μg, bold solid line; 0.1 μg, bold dashed line; 0.02 μg, thin dashed line) or left untreated (thin solid line). Arrows indicate rmIL-12 administration. Statistical evaluation by Student's *t*-test. $^*P = 0.13$, $^{**}P = 0.072$, $^{***}P = 0.078$

© Cancer Research Campaign 1998

### Cell staining and flow cytometry of lymphocytes

The spleen and cervical lymph node (CLN) of MG mice which had been treated with rmIL-12 at 0.5 μg i.p. were immediately removed after the animals were killed. Untreated MG mice were used as controls. The CLN and spleen were suspended mechanically in PBS. Cells remaining after the lysing of red blood cells were washed twice and counted (Hashimoto et al, 1995). Spleen cells ($10^6$) were incubated with FITC- or PE-conjugated MAb against mouse lymphocyte antigens CD3, CD4 and CD8 (PharMingen), and were analysed by FACScan (Becton Dickinson, Mountain View, CA).

### IFN-γ and IL-10 secretion

Lymphocytes ($10^6$) from the CLN and splenocytes ($10^6$) were cultured in 250 μl of complete medium (10% heat-inactivated FBS, 50 μM 2-ME, 20 mM HEPES, 110 μg $ml^{-1}$ sodium pyruvate, 2 mM L-glutamate and approximate concentrations of non-essential amino acids in RPMI1640) for 48 h. The culture medium were then collected and stored at –20°C until measurement of IFN-γ and IL-10 concentrations using ELISA kits purchased from Genzyme (Cambridge, MA, USA).

### Tumour rechallenge in MG mice cured by IL-12

RSV-M glioma cells ($5 \times 10^6$) or syngeneic C3H/MCA clone 15 fibrosarcoma cells ($5 \times 10^6$) were reinoculated into the cisterna magna of five MG mice which had received rmIL-12 i.p. and survived over 8 weeks. Other long-surviving MG mice after rmIL-12 i.p. were reinoculated subcutaneously (s.c.) with $10^7$ RSV-M glioma cells or C3H/MCA clone 15 fibrosarcoma cells. Diameters of s.c. tumours were measured with callipers, and the volume was calculated using the formula: volume = (longest diameter) × (shortest diameter)$^2$ × 1/2. All mice were 15–18 weeks old at the second tumour challenge.

**Figure 3** Anti-tumour effect of IL-12 against glioma in T-cell-deficient mice. BALB/c-nu/nu mice were treated with rmIL-12 at 0.5 μg i.p. once daily for 5 days (bold line) or left untreated (thin line). Statistical evaluation by Student's t-test. *$P = 0.17$

**Figure 4** Representative histology and immunohistochemistry of tumours and surroundings. Mice were killed 6 days after the inoculation of RSV-M glioma cells; the treated group was given rmIL-12 at 0.5 μg i.p. once daily beginning on day 3 after tumour inoculation. (**A**) Tumour area in an untreated mouse (H and E staining; magnification × 200), showing few lymphocytes. (**B**) Tumour area in a mouse treated with IL-12 at 0.5 μg i.p. (× 200), many infiltrating lymphocytes and a few necrotic areas seen in and around the tumour. (**C**) Immunohistochemically stained section adjacent to **B**, using MAb directed against CD4 (× 200), with many CD4+ lymphocytes being visible. **D**: Immunohistochemistry of section adjacent to B, using mAb directed against CD8 (× 200), with many CD8+ lymphocytes being seen. The T4/T8 ratio was approximately 0.4–0.6

© Cancer Research Campaign 1998

### RESULTS

### Tumour formation was observed in the CNS of MG mice

Three days after i.t. tumour inoculation (Figure 1A), glioma cells were frequently observed in the basal cisterns and ventricles and on the brain surface. Mass formation was observed in the basal cistern after 8 days (Figure 1B). Hydrocephalus was seen in several mice. Untreated mice lost body weight, developed paraplegia and were expected to die within 2 weeks after tumour inoculation.

### Intraperitoneal administration of IL-12 was effective in immunocompetent MG mice but not in T-cell-deficient MG mice

All untreated MG mice showed MG symptoms described in Materials and methods within 8 days (Figure 2A, thin solid line). The symptom-free time was prolonged in MG mice treated with IL-12 at 0.1 µg i.p., and 40% of these mice did not reveal any symptoms ($P$ = 0.053; Figure 2A, dashed line) for longer than 30 days. Eighty per cent of MG mice treated with IL-12 at 0.5 µg i.p. had been without MG symptoms for longer than 30 days ($P < 0.01$; Figure 2A, bold solid line), and they were judged to be cured. The median symptom-free time was not prolonged in any of the groups treated i.t. in comparison with the control group ($P > 0.07$; Figure 2B), although a few mice had been without MG symptoms for longer than 30 days in both the IL-12 at 0.1 and the IL-12 at 0.05-µg i.t. groups. These results showed that the local administration of IL-12 into the CNS was less effective in vivo than the systemic administration.

In BALB/c-nu/nu mice, in which NK activity is considered to be strong, all MG mice showed MG symptoms within 9 days both in the untreated group and in the IL-12 i.p. group ($P$ = 0.17; Figure 3). These findings revealed that i.p. IL-12 was effective in immunocompetent MG mice but not effective in T-cell-deficient MG mice. Furthermore, NK cells alone, without T cells, may not be capable of rejecting tumour cells transplanted into the CNS.

### $CD4^+$ and $CD8^+$ lymphocytes primarily infiltrated the tumour-bearing areas of MG mice after IL-12 treatment

Tumour formation and hydrocephalus were observed in brain sections of untreated mice, with tumour masses daily increasing in size (Figure 1). Few lymphocytes were present in or around the tumours of untreated mice (Figure 4A). In the MG mice treated with IL-12 i.p., tumours gradually increased in size from day 4 to day 6, but then became smaller on day 8, and no tumours were found on day 16 (data not shown). TIL and some necrotic regions were observed on day 6 in tumour areas of the MG mice treated with IL-12 i.p. (Figure 4B). These lymphocytes consisted mostly of $CD4^+$ and $CD8^+$ cells (Figure 4C and D), in a respective ratio of approximately 0.4–0.6. These results indicated that i.p. administration of IL-12 stimulates $CD4^+$ and $CD8^+$ lymphocytes to infiltrate into and around the tumours of MG mice.

**Figure 5** T lymphocyte populations in CLN and spleen of MG mice treated with i.p. IL-12 from days 3 to 7 (grey bars) and from days 3 to 13 (white bars) and of untreated controls (black bars). (**A–C**) Total numbers of CD3-, CD4- and CD8-positive lymphocytes, respectively, in the spleens. (**D–F**) Total numbers of CD3-, CD4- and CD8-positive lymphocytes, respectively, in the CLN. Bars indicate standard error (s.e.). Statistical evaluation by Student's *t*-test. $^*P < 0.05$, $^{**}P < 0.01$

© Cancer Research Campaign 1998

**Figure 6** IFN-γ and IL-10 production by lymphocytes in CLN and spleen of MG mice receiving IL-12 from days 3 to 7 (grey bars) and of untreated controls (black bars). Lymphocytes ($10^6$) were incubated in 250 μl of medium for 48 h and each cytokine was measured using an ELISA kit. (**A**) Concentration of IFN-γ produced by splenocytes. (**B**) Concentration of IL-10 produced by splenocytes. (**C**) Concentration of IFN-γ produced by CLN lymphocytes. (**D**) Concentration of IL-10 produced by CLN lymphocytes. Bars indicate s.e. Statistical evaluation by Student's *t*-test. $^*P < 0.05$, $^{**}P < 0.01$, ND not detected

### Numbers of CD3-, CD4- and CD8-positive T lymphocytes in CLN significantly increased in IL-12-treated MG mice

IL-12 did not have a significant effect on the cell distribution (CD3, CD4 and CD8) in the spleen and CLN of MG mice (data not shown). However, because the spleen and CLN were enlarged in i.p. IL-12-treated MG mice, the total number of T lymphocytes also increased as a natural consequence. Although the total numbers of CD3 (Figure 5A), CD4 (Figure 5B) and CD8 cells (Figure 5C) in the spleen of i.p. IL-12-treated MG mice (grey bars) were larger than in untreated mice (black bars), the increase was not statistically significant. T-cell population in the CLN of i.p. IL-12-treated MG mice significantly increased in comparison with untreated MG mice (Figures 5D–F). These MG models were then administered IL-12 i.p. from days 3 to 13 to compare the results with those in the mice treated for 5 days. Prolonged IL-12 treatment did not further affect the T-cell markers (Figure 5, white bars). Each T-cell population showed a maximum on day 8 in the spleen and on day 4 in the CLN.

### IFN-γ production by lymphocytes in the CLN was markedly increased in IL-12-treated MG mice

We examined IFN-γ and IL-10 production by lymphocytes in the CLN and spleen in MG mice with or without IL-12 treatment. The splenocytes obtained from i.p. IL-12-treated MG mice secreted the same level of IFN-γ as those from untreated mice (Figure 6A). On the other hand, a significant increase in IFN-γ was consistently observed in the lymphocytes derived from the CLN of i.p. IL-12-treated MG mice (Figure 6C). Although the splenocytes from treated MG mice secreted less IL-10 on days 6 and 8 than those from untreated mice (Figure 6B), the lymphocytes in the CLN obtained from treated MG mice secreted more IL-10 on days 4 and 6 than those from untreated mice (Figure 6D). In treated MG mice, lymphocytes from the CLN maintained a high level of IFN-γ secretion until day 6 and then production decreased on day 8, but IL-10 production was already decreased on day 6 (Figs 6C and D grey bars).

### MG mice cured by IL-12 rejected rechallenge with glioma cells

MG mice cured by IL-12 and surviving more than 8 weeks after the first inoculation of glioma cells were reinoculated with $5 \times 10^6$ glioma cells injected into the cisterna magna. These survivors had shown no MG symptoms without any treatment for more than 8 weeks after reinoculation with glioma cells ($P < 0.01$; Figure 7A). Apparent tumours were observed s.c. after 1 week when $1 \times 10^7$ glioma cells were inoculated s.c. into untreated mice (Figure 7B, black squares), but MG mice cured by IL-12 rejected these s.c. inoculated glioma cells completely ($P < 0.01$; Figure 7B, black circles). Syngeneic C3H/MCA clone 15 fibrosarcoma cells, however, grew s.c. larger in cured MG mice (Figure 7B, white circles).

 © Cancer Research Campaign 1998

**Figure 7** Rejection of the rechallenged glioma cells both intrathecally (**A**) or subcutaneously (**B**) in cured MG mice by IL-12. Statistical evaluation by Student's *t*-test. $^*P < 0.01$. (**A**) MG mice cured by intraperitoneal (i.p.) IL-12 were rechallenged with the same RSV-M glioma cells intraperitoneally (i.t.). All reinoculated MG mice remained without MG symptoms for longer than 8 weeks (bold line), while all MG mice inoculated for the first time had shown MG symptoms within 9 days after a single inoculation (thin line). (**B**) Growth of subcutaneous (s.c.) transplanted RSV-M glioma cells and C3H/MCA clone 15 fibrosarcoma cells. While growth of the s.c. transplanted tumour cells was observed in normal mice (black squares), MG mice which had been cured by IL-12 subsequently rejected the same cells when transplanted s.c. (black circles). C3H/MCA clone 15 fibrosarcoma cells grew up both in cured MG mice (white circles) and in normal mice (white squares). Bars indicate s.e.

### DISCUSSION

In this report, we applied the MG model to investigate the immunological response against tumour cells in the CNS. Although the pia mater separates subarachnoid space from brain parenchyma, some intrathecally injected glioma cells show parenchymal invasion (Kitamura et al, 1996; unpublished data); in addition, glioma cells undergo CSF pathway dissemination in animal models and in patients with advanced glioma (Arita et al, 1994; Yamada et al, 1994). On the other hand, the BBB or the blood–CSF barrier around the tumour mass in the CNS was thought to be destroyed, and thus tumour antigens gained access to the systemic immune system via tumour vessels or Virchow–Robin space, which continues to subarachnoid space. Further, it was easy to carry out the MG model on mice, and the appearance of MG symptoms showed good correlation with the numbers of intrathecally injected tumour cells. In this way, we used the MG model to investigate the anti-tumour efficacy of IL-12 in the CNS.

In this paper, we have demonstrated that systemic administration of IL-12 was surprisingly effective against malignant tumours in the subarachnoid space of the CNS (MG mice). As previously reported (Tahara et al, 1994), IL-12 does not directly affect the growth of RSV-M glioma cells in vitro (data not shown). However, systemic IL-12 prolonged the symptom-free time in the present study ($P < 0.01$; Figure 2A). Not only systemic administration but also local or peritumoral injection of IL-12 has been reported to induce the regression of some subcutaneous tumours (Brunda et al, 1993; Tahara et al, 1994). Although i.t. IL-12 treatment was less effective when judged on the basis of the median symptom-free time ($P > 0.07$; Figure 2B), some MG mice were nevertheless cured by i.t. administration. A large i.t. dose of IL-12 may have some toxicity because some normal mice had lost body weight during and after i.t. administration of 0.5 μg of IL-12 (data not shown). As treatment with IL-12 at 0.5 μg i.t. induced splenomegaly, as did i.p. treatment (data not shown), i.t. IL-12 would affect systemic immunity. These findings suggest that i.t. injection of IL-12 has some anti-tumour effect, but that its toxicity offsets its efficacy. Thus, systemic IL-12 may be the preferable choice even for the treatment of CNS tumours.

Under normal conditions, no lymphocytes are present in the brain parenchyma. TIL, however, are found in and around the tumours in 35–80% of glioma patients (de Micco, 1989). B lymphocytes and NK cells do not play major roles in suppressing the growth of human gliomas (Sawamura et al, 1988). TIL are activated by IL-12 in vitro (Andrews et al, 1993), and IL-12 can induce the production of $CD8^+$ CTL in vitro (Bloom et al, 1994). Large numbers of TIL were observed in immunocompetent MG mice treated with IL-12 (Figure 4), and IL-12 did not show anti-tumour activity in T-cell-deficient mice ($P = 0.17$; Figure 3). RSV-M glioma cells were rejected when they were reinoculated i.t. or s.c. into MG mice cured by IL-12 ($P < 0.01$; Figure 7), but syngeneic C3H/MCA clone 15 fibrosarcoma cells were not rejected (Figure 7). These findings indicate that tumour-specific immunity is induced systemically and/or in the CNS of MG mice cured by IL-12, and that T lymphocytes produced as a result of activation by IL-12 act as major anti-tumour effectors in MG mice.

Many tumour antigens in the CNS are mainly phagocytized by microglia (Shrikant et al, 1996), and a significant percentage of the CSF drains into the CLN (Kida et al, 1993, 1995; Cserr et al, 1992). In MG models, tumour antigens would reach the CLN via this route. In the CLN, APC activate Th cells and CTL, and they secrete a moderate amount of IFN-γ. Systemic administration of IL-12 also activates Thl cells and CTL (Seder et al, 1993; Kennedy et al, 1994; Nastala et al, 1994). In the CLN from IL-12-treated MG mice, a large number of T lymphocytes and a high level of IFN-γ production were detected (Figures 5 and 6). IFN-γ production by lymphocytes in the CLN from IL-12-treated MG mice was markedly increased ($P < 0.01$; Figure 6C), and it was

© Cancer Research Campaign 1998 

higher than that in the CLN from normal mice treated with IL-12 (data not shown). These findings suggest that both systemic IL-12 and tumour antigens are necessary for a high level of IFN-γ secretion. The expression of MHC antigens can be induced in both glial and glioma cells in the CNS by IFN-γ (Wong et al, 1984; de Micco, 1989; Tamura et al, 1989). Activated glial cells produce granulocyte–macrophage colony-stimulating factor (Ohno et al, 1990; Lee et al, 1994), and this factor activates microglia, which are one type of APC in the CNS (Frei et al, 1987; Matsumoto et al, 1992; Shrikant et al, 1996). In this way, although it has been thought that the presence of the BBB and the absence of lymphatic tissues segregate the CNS from systemic immunity, the immune system would appear to recognize the tumour antigens in the subarachnoid space of the CNS, and IL-12 may activate immunological reactions against tumours in the CNS. Furthermore, Th cells and CTL also efficiently infiltrate tumours in the CNS.

Although it is well known that IL-12 stimulates T and NK cells to secrete IFN-γ, there is relatively little information on the effect of IL-12 on T cells for production of IL-10. It has been reported that in vivo treatment of IL-12 increases in both IFN-γ and IL-10 mRNA and that the presence of IL-12 results in an efficient priming of the clones for high production of both IFN-γ and IL-10 (Finkelman et al, 1994; Gerosa et al, 1996). In the present study, both IFN-γ and IL-10 production elevated on days 4 and 6 in the CLN from IL-12 treated MG mice (Figure 6D). Elevation of IL-10 is supposed to affect an anti-inflammatory response involving down-regulation of Th1 (Gerosa et al, 1996). Some investigators have, however, reported that production of IL-10 in bulk T-cell cultures stimulated in vitro with allergen is inhibited if IL-12 is added to the cultures (Marshall et al, 1995). The other investigators have reported that the IL-10-producing clone is primed only if IL-12 is added to the culture in the first few days (Gerosa et al, 1996). In this study, IL-10 levels of the CLN decreased by day 6 and were not detected on day 8, while IFN-γ production was still maintained at a high level on day 6 and present on day 8 (Figures 6C and D). The release of the hyperimmune state and/or the prolonged stimulation of IL-12 might result in the decrease of IL-10 level preceding that of IFN-γ level.

The results of this study indicate that systemic IL-12 administration induces T-lymphocyte-mediated immune responses in the CNS, and that CTL appear to play an important role in the rejection of glioma cells in the CNS. Furthermore, IL-10 secretion may suppress such inflammatory functions in these models.

### ACKNOWLEDGEMENT

This work was supported by a Grant-in-Aid for Scientific Research (08457363) from the Ministry of Education, Science, Sports and Culture of Japan.

### REFERENCES

Andrews JVR, Schoof DD, Bertagonalli MM, Peoples GE, Goedegebuure PS and Eberlein TJ (1993) Immunomodulatory effects of interleukin-12 on human tumor-infiltrating lymphocytes. *J Immunother* **14**: 1–10

Annegers JF, Schoenberg BS, Okazaki H and Kurland LT (1981) Epidemiologic study of primary intracranial neoplasm. *Arch Neurol* **38**: 217–219

Arita N, Taneda M and Hayakawa T (1994) Leptomeningeal dissemination of malignant gliomas. Incidence, diagnosis and outcome. *Acta Neurochir* **126**: 84–92

Barba D, Saris SC, Holder C, Rosenberg SA and Oldfield EH (1989) Intratumoral LAK cell and interleukin-2 therapy of human gliomas. *J Neurosurg* **70**: 175–182

Bloom ET and Horvath JA (1994) Cellular and molecular mechanism of the IL-12-induced increase in allospecific murine cytolytic T cell activity. *J Immunol* **152**: 4242–4254

Brunda MJ (1994) Interleukin-12. *J Leukoc Biol* **55**: 280–288

Brunda MJ and Gately MK (1994) Antitumor activity of interleukin-12. *Clin Immunol Immunother* **71**: 253–255

Brunda MJ, Luistro L, Warrier RR, Wright RB, Hubbard BR, Murphy M, Wolf SF and Gately MK (1993) Antitumor and antimetastatic activity of interleukin 12 against murine tumors. *J Exp Med* **178**: 1223–1230

Cesano A, Visonneau S and Santoli D (1995) Treatment of experimental glioblastoma with a human major histocompatibility complex non-restricted cytotoxic T cell line. *Cancer Res* **55**: 96–101

Chan SH, Perussia B, Gupta JW, Kobayashi M, Pospísil M, Young HA, Wolf SF, Young D, Clark SC and Trinchieri G (1991) Induction of interferon γ production by natural killer cell stimulatory factor: characterization of the responder cells and synergy with other inducers. *J Exp Med* **173**: 869–879

Cserr HF, Harling Berg CJ and Knopf PM (1992) Drainage of brain extracellular fluid into blood and deep cervical lymph and its immunological significance. *Brain Pathol* **2**: 269–276

de Micco C (1989) Immunology of central nervous system tumors. *J Neuroimmunol* **25**: 93–108

Färkkilä M, Jääskeläinen J, Kallio M, Blomstedt G, Raininko R, Virkkunen P, Paetau A, Sarelin H and Mäntrylä M (1994) Randomized, controlled study of intratumoral recombinant γ-interferon treatment in newly diagnosed glioblastoma. *Br J Cancer* **70**: 138–141

Finkelman FD, Madden KB, Cheever AW, Katona IM, Morris SC, Gately MK, Hubbard BR, Gause WC and Urban Jr. JF (1994) Effect of interleukin 12 on immune response and host protection in mice infected with intestinal nematode paresis. *J Exp Med* **179**: 1563–1572

Fontana A, Hengatner H, de Tribolet N and Weber E (1984) Glioblastoma cells release interleukin 1 and factors inhibiting interleukin 2-mediated effects. *J Immunol* **132**: 1837–1844

Fontana A, Frei K, Bodmer S and Hofer E (1987) Immune-mediated encephalitis: on the role of antigen-presenting cells in brain tissue. *Immunol Rev* **100**: 185–201

Frank E, Pulver M and de Tribolet N (1986) Expression of class II major histocompatibility antigens on reactive astrocytes and endothelial cell within the gliosis surrounding metastases and abscesses. *J Neuroimmunol* **12**: 29–36

Frei K, Siepl C, Groscurth P, Bodmer S, Schwerdel C and Fontana A (1987) Antigen presentation and tumor cytotoxicity by interferon-γ-treated microglial cells. *Eur J Immunol* **17**: 1271–1278

Gately MK, Warrier RR, Honasoge S, Carvajal DM, Faherty DA, Connaughton SE, Anderson TD, Sarmiento U, Hubbard BR and Murphy M (1994) Administration of recombinant IL-12 to normal mice enhances cytolytic lymphocyte activity and induces production of IFN-γ *in vivo*. *Int Immunol* **6**: 157–167

Gerosa F, Paganin C, Peritt D, Paiola F, Scupoli MT, Aste-Amezaga M, Frank I and Trinchieri G (1996) Interleukin-12 primes human CD4 and CD8 T cell clones for high production of both interferon-gamma and interleukin-10. *J Exp Med* **183**: 2559–2569

Hashimoto W, Takeda K, Anzai R, Ogasawara K, Sakihara H, Sugiura K, Seki S and Kumagai K (1995) Cytotoxic NK1.1 $Ag^+$ α β T cells with intermediate TCR induced in the liver of mice by IL-12. *J Immunol* **154**: 4333–4340

Hickey WF, Vass K and Lassmann H (1992) Bone marrow-derived elements in the central nervous system: an immunohistochemical and ultrastructural survey of rat chimeras. *J Neuropathol Exp Neurol* **51**: 246–256

Kennedy MK, Picha KS, Shanebeck KD, Anderson DM and Grabstein KH (1994) Interleukin-12 regulates the proliferation of Th1, but not Th2 or Th0, clones. *Eur J Immunol* **24**: 2271–2278

Kida S, Pantazis A and Weller RO (1993) CSF drains directly from the subarachnoid space into nasal lymphatics in the rat. Anatomy, histology and immunological significance. *Neuropathol Appl Neurobiol* **19**: 480–488

Kida S, Weller RO, Zhang E-T, Phillips MJ and Iannotti F (1995) Anatomical pathways for lymphatic drainage of the brain and their pathological significance. *Neuropathol Appl Neurobiol* **21**: 181–184

Kitamura I, Kochi M, Matsumoto Y, Ueoka R, Kuratsu JI and Ushio Y (1996) Intrathecal chemotherapy with 1,3-bis(2-chloroethyl)-1-nitrosourea encapsulated into hybrid liposome for meningeal gliomatosis: an experimental study. *Cancer Res* **56**: 3986–3992

Kobayashi M, Fitz L, Ryan M, Hewick RM, Clark SC, Loudon R, Sherman F, Perussia B and Trinchieri G (1989) Identification and purification of natural killer cell stimulatory factor (NKSF), a cytokine with multiple biologic effects on human lymphocytes. *J Exp Med* **170**: 827–845

 © Cancer Research Campaign 1998

Kumanishi T, Ikuta F and Yamamoto T (1973) Brain tumors induced by Rous sarcoma virus, Schmidt-Ruppin stain. III. Morphology of brain tumors induced in adult mice. *J Natl Cancer Inst* **51**: 95–109

Lee SC, Lic W, Brosan CF and Dickson DW (1994) GM-CSF promotes proliferation of human fetal and adult microglia in primary cultures. *Glia* **12**: 309–318

Lillehei KO, Mitchell DH, Johnson SD, McCleary EL and Kruse CA (1991) Long-term follow up of patients with recurrent malignant gliomas treated with adjuvant adoptive immunotherapy. *Neurosurgery* **28**: 16–23

Marshall JD, Secrist H, DeKruyff RH, Wolf SF and Umetsu DT (1995) IL-12 inhibits the production of IL-4 and IL-10 in allergen-specific human CD4+ T lymphocyte. *J Immunol* **155**: 111–117

Martinotti A, Stoppacciaro A, Vagliani M, Melani C, Spreafico F, Wysocka M, Parmiani G, Trinchieri G and Colombo MP (1995) CD4 T cells inhibit in vivo the CD8-mediated immune response against murine colon carcinoma cells transduced with interleukin-12 genes. *Eur J Immunol* **25**: 137–146

Matsumoto Y, Ohmori K and Fujiwara M (1992) Immune regulation by brain cells in the central nervous system: microglia but not astrocytes present myelin basic protein to encephalitogenic T cell under *in vivo*-mimicking condition. *Immunology* **76**: 209–216

Mu J, Zou J-P, Yamamoto N, Tsutsui T, Tai X-G., Kobayashi M, Herrmann S, Fujiwara H and Hamaoka T (1995) Administration of recombinant interleukin 12 prevents outgrowth of tumor cells metastasizing spontaneously to lung and lymph nodes. *Cancer Res* **55**: 4404–4408

Nastala CL, Edington HD, McKinney TG, Tahara H, Nalesnik MA, Brunda MJ, Wolf SF, Gately MK, Schreiber RD, Storkus WJ and Lotze MT (1994) Recombinant IL-12 administration induces tumor regression in association with IFN-γ production. *J Immunol* **153**: 1697–1706

Nazzaro JM and Neuwelt EA (1990) The role of surgery in the management of supratenotrial intermediate and high-grade astrocytoma in adults. *J Neurosurg* **73**: 331–344

Nitta T, Sato K, Yagita H, Okumura K and Ishii S (1990) Preliminary trial of specific targeting therapy against malignant glioma. *Lancet* **335**: 368–376

Ohno K, Susumura A, Sawada M and Marunouchi T (1990) Production of granulocyte/macrophage colony-stimulating factor by cultured astrocytoma. *Biochem Biophys Res Commun* **169**: 719–724

Rapp UR, Nowinski RC, Reznikoff CA and Heidelberger C (1975) Endogenous oncornaviruses in chemically induced transformant. *Virology* **65**: 392–409

Sawamura Y, Abe H, Aida T, Hosokawa M and Kobayashi H (1988) Isolation and *in vitro* growth of glioma-infiltrating lymphocytes, and an analysis of surface phenotypes. *J Neurosurg* **69**: 745–750

Schoenhaut DS, Chua AO, Wolitzky AG, Quinn PM, Dwyer CM, McComas W, Familletti PC, Gately MK and Gubler U (1992) Cloning and expression of murine IL-12. *J Immunol* **148**: 3433–3440

Seder RA, Gazzinelli R, Sher A and Paul WE (1993) Interleukin 12 acts directly on CD4+ T cells to enhance priming for interferon γ production and diminishes interleukin 4 inhibition of such priming. *Proc Natl Acad Sci USA* **90**: 10188–10192

Shimizu K, Okamoto Y, Miyao Y, Yamada M, Ushio Y, Hayakawa T, Ikeda H and Mogami H (1987) Adoptive immunotherapy of human meningeal gliomatosis and carcinomatosis with LAK cells and recombinant interleukin-2. *J Neurosurg* **66**: 519–521

Shrikant P and Benveniste N (1996) The central nervous system as an immunocompetent organ. *J Immunol* **157**: 1819–1821

Stern AS, Podlasky FJ, Hulmes JD, Pan YCE, Quinn PM, Wolitzky AG, Familletti PC, Stremlo DL, Truitt T, Chezzonite R and Gatel MK (1990) Purification to homogeneity and partial characterization of cytotoxic lymphocyte maturation factor from human B-lymphoblastoid cells. *Proc Natl Acad Sci USA* **87**: 6808–6812

Tahara H and Lotze MT (1995) Antitumor effects of interleukin-12 (IL-12): applications for the immunotherapy and gene therapy of cancer. *Gene Therapy* **2**: 96–106

Tahara H, Zeh III HJ, Storkus WJ, Pappo I, Waltkins SC, Gubler U, Wolf SF, Robbins PD and Lotze MT (1994) Fibroblasts genetically engineered to secrete interleukin 12 can suppress tumor growth and induce antitumor immunity to murine melanoma *in vivo*. *Cancer Res* **54**: 182–189

Tamura K, Shimizu K, Yamada M, Okamoto Y, Matsui Y, Park KC, Mabuchi E, Moriuchi S and Mogami H (1989) Expression of major histocompatibility complex on human medulloblastoma cells with neuronal differentiation. *Cancer Res* **49**: 5380–5384

UKCCR (1988) UKCCR guidelines for the welfare of animals in experimental neoplasia. *Br J Cancer* **58**: 109–113

Wolf SF, Temple PA, Kobayashi M, Young D, Dicig M, Lowe L, Dzialo R, Fitz L, Ferenz C, Hewick RM, Kelleher K, Herrmann SH, Clark SC, Azzoni L, Chng SH, Trinchieri G and Perussia B (1991) Cloning of cDNA for natural killer cell stimulatory factor, a heterodimeric cytokine with multiple biologic effects on T and natural killer cells. *J Immunol* **146**: 3074–3081

Wong GHW, Bartlett PF, Clark-Lewis I, Battye F and Schrader JW (1984) Inducible expression of H-2 and Ia antigens on brain cells. *Nature* **310**: 688–691

Yamada M, Shimizu K, Miyao Y, Hayakawa T, Nakajima K, Nakahira K, Nakagawa H, Mikoshiba K and Ikenaka K (1994) Migration of genetically labeled glioma cells after implantation into murine brain. *J Neurosci Res* **38**: 415–423

Yoshida J, Kajita Y, Wakabayashi T and Sugita K (1994) Long-term follow-up results of 175 patients with malignant glioma: importance of radical tumor resection and postoperative adjuvant therapy with interferon, ACNU and radiation. *Acta Neurochir* **127**: 55–59

Zitvogel L, Robbins PD, Storkus WJ, Clarke MR, Maeurer MJ, Campbell RL, Davis CG, Tahara H, Schreiber RD and Lotze MT (1996) Interleukin-12 and B7.1 co-stimulation cooperate in the induction of effective antitumor immunity and therapy of established tumors. *Eur J Immunol* **26**: 1335–1341

© Cancer Research Campaign 1998